# Posterior cruciate ligament resection increases intraoperative lateral and medial flexion laxity during total knee arthroplasty

**DOI:** 10.1007/s00264-025-06413-7

**Published:** 2025-01-28

**Authors:** Nathan Alloun, Alexander Orsi, Christopher Plaskos, Thomas Brosset, Florian Boureau, Sophie Putman

**Affiliations:** 1 University of Lille, 42 rue Paul Duez, 59000 Lille, Nord France; 2 Corin, Raynham, MA USA; 3 Fonvert Clinique, Sorgues, Vaucluse France; 4 Metrics, CHU de Lille, Nord, France

**Keywords:** Posterior cruciate ligament (PCL) retention, PCL sacrifice, Total knee arthroplasty, Gap balancing, Alignment

## Abstract

**Purpose:**

This study reports the relationship between posterior cruciate ligament (PCL) retention vs resection and soft tissue laxity and balance throughout flexion using a robotically controlled ligament tensioner.

**Methods:**

55 robotic-assisted TKAs (Total knee arthroplasty) were retrospectively reviewe. The robotic ligament tensioner collected laxity data both before and after PCL resection. Medial and lateral coronal laxity were compared before and after PCL resection at 10°, 45°, and 90° flexion. Gap opening was compared between pre-operative coronal hip-knee-ankle groups.

**Results:**

Lateral laxity was greater after PCL resection at 60° (12.7 ± 2 vs 11.5 ± 3 mm), 75° (13.2 ± 2 vs 11.8 ± 3 mm), and 90° (13.7 ± 2 vs 12.1 ± 3 mm). Medial laxity was significantly greater after PCL resection at 90° (10.1 ± 2 vs 9 ± 2 mm). After PCL resection, laxity in valgus knees increased more compared to neutral/varus knees laterally at 30° (1.2 ± 1 vs 0.3 ± 1 mm), 45° (1.6 ± 1 vs 0.6 ± 1 mm), and 60° (2.1 ± 2 vs 1 ± 1 mm). A similar, but non-significant trend was observed at 90° (2.7 ± 2 vs 1.5 ± 1 mm, p = 0.09).

**Conclusion:**

PCL resection increases flexion laxity laterally by up to 1.6 mm and medially by 1.1 mm on average, with valgus knees increasing more than neutral/varus knees. The findings emphasize that surgeons should consider the interplay between PCL resection and coronal deformity when planning and executing TKA procedures.

## Introduction

Resection of the posterior cruciate ligament (PCL) and its implications on gap balancing in total knee arthroplasty (TKA) continues to be debated. The general consensus is that PCL resection results in increased flexion laxity in TKA, with little effect on extension gaps; however, the degree of laxity increase in flexion and midflexion and the effect on gap symmetry is not consistent among reports [1–3]. Additionally, few studies to our knowledge have reported whether this relationship differs between varus and valgus knee deformities, which would have implications on soft tissue balancing in TKA [1, 4]. Prior studies have also been limited in that they have been performed in cadavers or small patient numbers or have used manual distraction methods or manual instruments and measurement gauges with limited accuracy [1–3, 5, 6].

The aim of this study was to therefore quantify the effect of PCL resection on tibiofemoral gaps using a constant distraction tension applied to the medial and lateral sides throughout the range of flexion. A secondary aim was to determine if pre-operative coronal deformity affected the relationship between tibiofemoral gap symmetry in the PCL resected vs retained knee. It is hypothesized that the flexion gap will increase after PCL resection, with the lateral flexion space opening more than the medial on average, and that valgus knees will experience greater opening laterally in flexion than neutral/varus knees.

## Methods

### Patients

55 robotic-assisted TKAs were retrospectively reviewed from two surgeons operating at two institutions between November 2022 to April 2024 following institutional review board approval (WCG-IRB no. 120190312). All operations were performed using the OMNIBotics system with the BalanceBot robotic ligament tensioning tool (Corin, Raynham, MA, USA) and the HLS KneeTec total knee implant system (Corin, Cirencester, UK). Inclusion criteria included cases where tibiofemoral gaps were acquired with the robotic ligament tensioning tool after tibial resection but before femoral resection, both prior to and after resecting the PCL. Exclusion criteria included patients with prior knee surgery (ligamentoplasty, osteotomy, prosthesis revision) and systemic inflammatory disease. Patient demographics are summarized in Table 1.

**Table 1:** Patient demographics, mean ± standard deviation, [range]

Pre-operative parameter	Value
Age (years)	70.7 ± 89 [54 – 87]
Sex (female/male)	35/20
BMI	29.7 ± 5.1 [19.6 – 43.5]
Coronal deformity (°, + ve = varus)	1.9 ± 6.3 [−15 – 18]

### Surgical technique

**Fig. 1:**
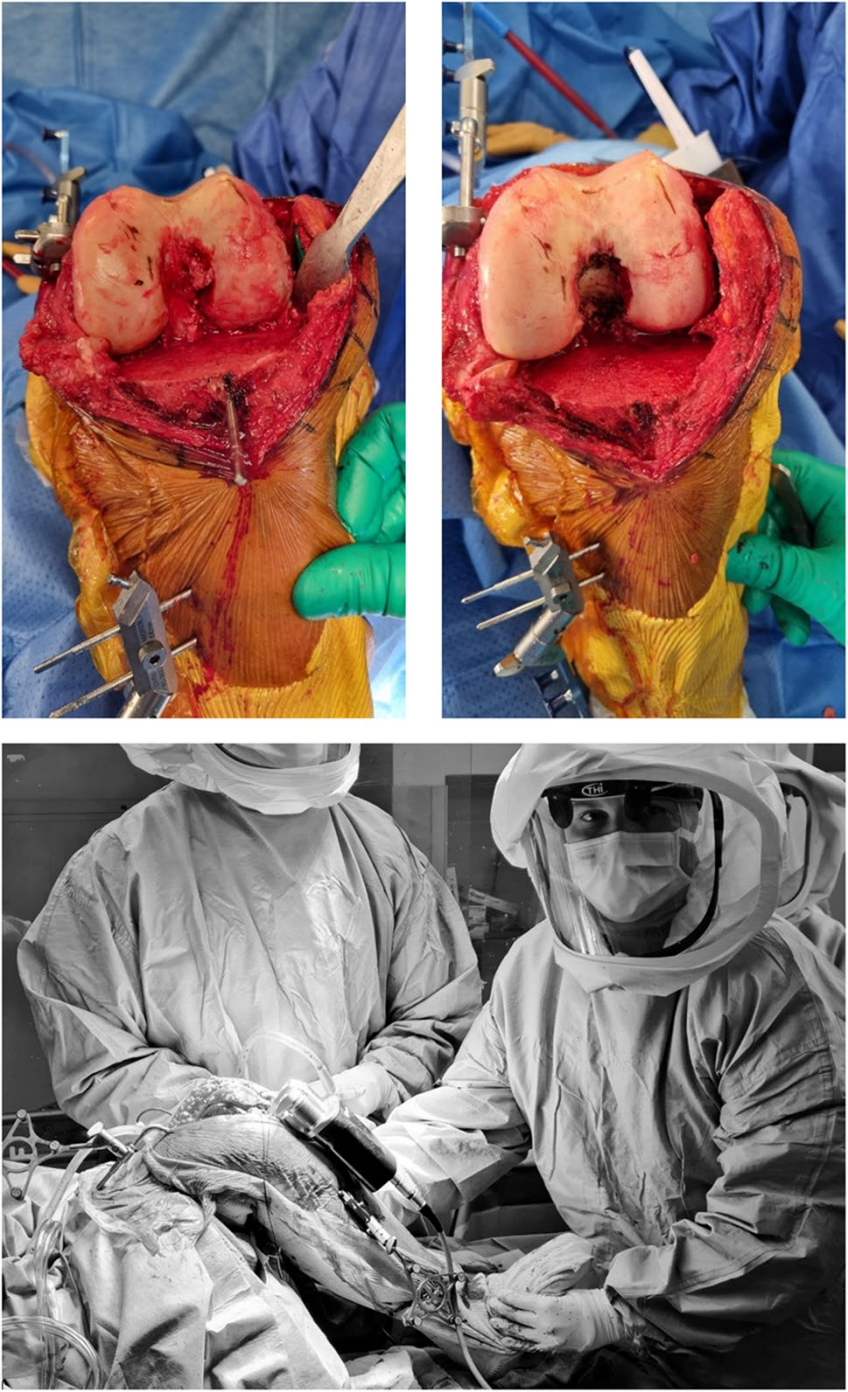
Intra-operative photographs showing knee before (upper left) and after (upper right) resection of the posterior cruciate ligament (PCL), and with the robotic tensioner inserted into the joint with the patella reduced (bottom)

**Fig. 2:**
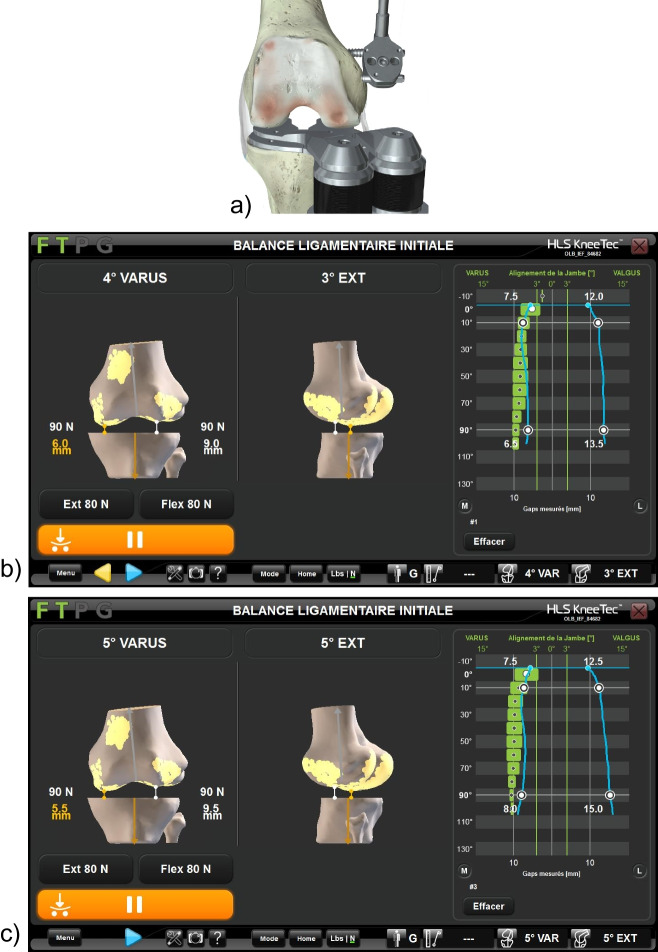
A robotic ligament tensioner collected laxity data before and after PCL resection (**a**). Screenshots show the gap acquisition throughout the range of motion (blue lines on right side of screen) before (**b**) and after (**c**) PCL resection for an example patient

The surgical technique was identical across both surgeons and used a medial parapatellar approach. The patella was everted during the approach but returned to its original position during the robotic gap acquisition. The medial collateral ligament was not released. The anterior cruciate ligament (ACL) was excised, and two pins were fixed in the femur and tibia medially to attach the robotic system trackers. After bone registration and initial kinematic assessment where the hip-knee-ankle (HKA) alignment and varus/valgus opening was recorded through the flexion range, a neutral tibial resection was performed within 2° varus while taking care to retain the PCL and associated tibial bone island during the resection, Fig. 1. This enabled acquisition of the joint gaps from 90° to 10° flexion before PCL release using the digital ligament tensor, Fig. 1 (bottom) and Fig. 2a,b. A second acquisition was then performed after PCL release, again from 90° to 10° flexion, Fig. 1 upper-right and Fig. 2c. The distraction force of the ligament tensor was set at 80N on the medial and lateral sides in both extension and flexion.

### Data analyses

Laxity is defined as the distance from the resected tibia to the femoral bone surface with the robotic ligament tensioner inserted and applying load. Gap opening was calculated as laxity before PCL resection minus laxity after PCL resection. Shapiro–Wilk tests were performed and reported non-normally distributed data. Medial and lateral coronal laxity were compared before and after PCL resection at 10°, 45°, and 90° of flexion using paired Wilcoxon ranked sum tests. Medial and lateral gap openings were also compared using paired Wilcoxon ranked sum tests at all flexion angles. Gap opening was compared between pre-operative coronal hip-knee-ankle (HKA) groups (> 2° Valgus: ‘Valgus’ [*n* = 12] and ≤ 2° Valgus: ‘Neutral/Varus’ [*n* = 43], as measured by the navigation system) using Wilcoxon ranked sum tests. All analysis was performed in R version 4.1.0 (R Foundation for Statistical Computing). A p-value < 0.05 was considered statistically significant.

An a-priori matched-pair power analysis for the difference in means for Wilcoxon ranked sum tests was performed using the following parameters: alpha = 0.05, beta = 0.8, difference in the means of 0.5 mm, and a standard deviation of the differences of 1 mm. G*Power (Heinrich-Heine-University, Düsseldorf, Germany) indicated the total sample size required would be 35.

## Results

### PCL intact vs resected, overall cohort

Lateral and medial laxity increased after PCL resection at 45° and 90° of flexion, Table 2. Lateral laxity increased more than medial laxity at 45° (0.8 ± 1.1 vs 0.4 ± 0.8 mm, *p* < 0.01), and 90° (1.7 ± 1.7 vs 1.2 ± 1.1 mm, *p* < 0.001).

**Table 2:** Effect of PCL resection on medial and lateral gaps (mean ± SD). ∆ indicates difference between PCL intact and resected. Bold *p*-values indicate *p* < 0.05

	Lateral				Medial				*p*-value comparing ∆ medial vs ∆ lateral
Flexion	Intact	Resected	∆	*p*-value	Intact	Resected	∆	*p*-value	
10°	10.4 ± 1.9	10.4 ± 2.1	0 ± 0.9	0.733	8.1 ± 2.2	8.3 ± 2.4	0.2 ± 0.7	0.061	0.061
45°	11.3 ± 2.4	12.1 ± 2.2	0.8 ± 1.1	** < 0.001**	9 ± 2.4	9.4 ± 2.5	0.4 ± 0.8	** < 0.001**	**0.002**
90°	12.1 ± 2.8	13.8 ± 2.3	1.7 ± 1.7	** < 0.001**	9 ± 2.1	10.2 ± 2.1	1.2 ± 1.1	** < 0.001**	**0.001**

### Neutral/varus vs valgus knees

**Fig. 3:**
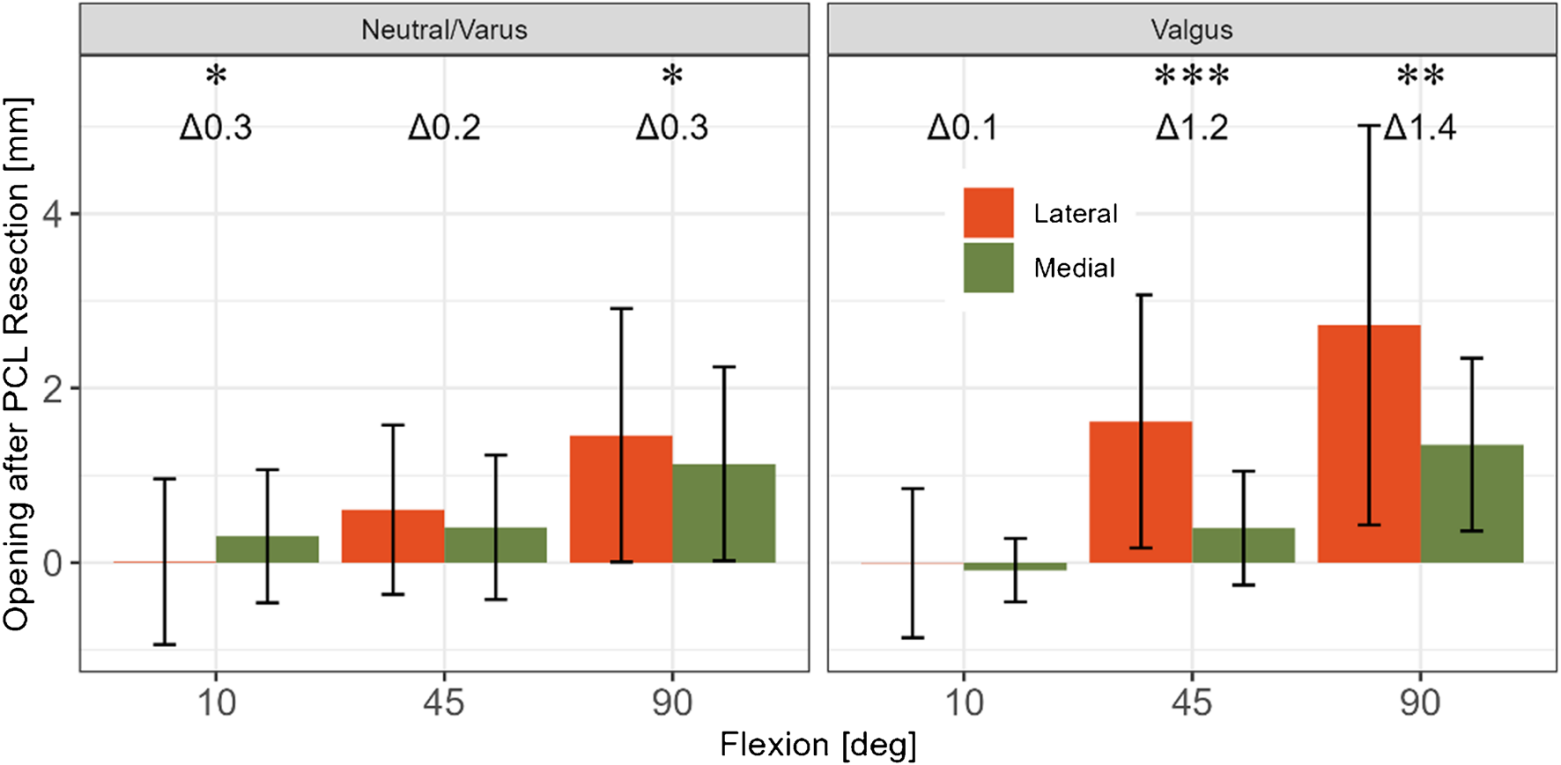
Effect of pre-operative coronal HKA on medial and lateral gap opening after PCL resection [mm]. * = *p* < 0.05, ** = *p* < 0.01, *** = *p* < 0.001

In neutral/varus knees, the gap opening was 0.3 mm larger laterally than medially at 10 and 90° flexion after PCL resection, Fig. 3. In valgus knees after PCL resection, the gap opening was 1.2 and 1.4 mm larger laterally than medially at 45 and 90° flexion, respectively. After PCL resection, lateral laxity in valgus patients increased more at 45° (1.6 ± 1 vs 0.6 ± 1 mm, *p* < 0.05) compared to the neutral/varus group, Table 3. A similar though not statistically significant result was observed at 90° (2.7 ± 2 vs 1.5 ± 1 mm, *p* = 0.110). No significant differences in medial laxity increases were observed between the valgus and neutral/varus groups.

**Table 3:** Medial and lateral gap opening after PCL resection by pre-operative coronal HKA, [mm] (mean ± SD). ∆ indicates mean difference between valgus and neutral/varus HKA. Bold *p*-values indicate *p* < 0.05

	Lateral				Medial			
Flexion	Valgus	Neutral/Varus	∆	*p*-value	Valgus	Neutral/Varus	∆	*p*-value
10°	0 ± 0.9	0 ± 1.0	0	0.872	−0.1 ± 0.4	0.3 ± 0.8	−0.4	0.138
45°	1.6 ± 1.4	0.6 ± 1.0	1	**0.036**	0.4 ± 0.7	0.4 ± 0.8	0	0.784
90°	2.7 ± 2.3	1.5 ± 1.5	1.2	0.110	1.4 ± 1.0	1.1 ± 1.1	0.3	0.465

## Discussion

The most important finding of this study is that PCL resection resulted in increased gap opening with increasing flexion, and that the amount of increase was associated with pre-operative coronal deformity. Specifically, PCL resection in neutral/varus knees resulted in gaps opening nearly symmetrically at 90° flexion (with only 0.4 mm mean difference between medial and lateral) while valgus knees had considerably larger opening laterally than medially by 1 and 1.2 mm at 45 and 90° flexion, respectively. Moreover, the opening laterally in flexion in valgus knees was larger and more variable at 2.7 ± 2.3 mm (mean ± SD) compared with 1.1 ± 1.1 for neutral/varus knees. The difference between valgus and neutral/varus knees has implications on knee soft tissue balancing in the context of PCL resection, and surgeons should expect greater opening laterally in midflexion and flexion when resecting the PCL in valgus knees.

Our overall results agree with those of Warth et al. who investigated the effect of PCL resection in 129 TKAs performed by two surgeons [1]. They found that the mean increase in joint space after PCL resection was 0.3 mm in extension, 1 mm at 45° flexion and 1.7 mm at 90° flexion. Although they found no statistically significant difference between the opening of the medial and lateral sides with pre-operative deformity, a trend nearing significance was reported for valgus knees to open up laterally in flexion (p = 0.079). This trend may have become statistically significant had more patients been included their study, or if a precision digital distraction instrument been used rather than a manual distraction device that did not allow for standardization of the distraction force on the medial and lateral sides [1]. Using optical tracking and manual varus and valgus stresses applied to the knee, another study found significantly larger lateral than medial flexion gap openings both overall and in pre-operative valgus knees [4]. Specifically, in a subgroup analysis of 14 valgus patients, Kayani et al. reported that the flexion gap opened more laterally than medially after PCL resection, by 1.9 and 3.1 mm, respectively, which is similar to the medial and lateral openings of 1.4 and 2.7 mm reported in our study for valgus knees. As shown by Iwakiri and al [7], valgus knee are a different population, resulting in different strategies.

Post-operative stiffness is a known complication following TKA and has been reported to occur in 2 to 16% of patients [8, 9]. Some authors recommend arthroscopic lysis with PCL release to treat knee stiffness and flexion deficit following TKA [10]. However, our results and those of others suggest PCL resection may produce variable and unpredictable results, with either limited gap opening or in some cases several millimeters of opening if the entire PCL is released (maximum opening after PCL release was 7 mm in flexion laterally in our study, which is similar to the maximum openings reported in other studies [1–3]). Therefore, caution should be exercised when releasing the PCL following TKA to treat a tight flexion gap so as not to create an instability, and surgeons should observing for PCL tightening and consider performing a partial release first while monitoring flexion gap opening. Moreover, when planning implant placement in TKA using gap referencing technique, if the PCL is intended to be resected, this should be performed prior to performing gap measurements and making bone resections to avoid having an overly large flexion gap after TKA.

A strength of our study over prior investigations is that our study used a digital tensioning device to standardize the load applied to knee across all patients combined with optical tracking technology which has been shown to produce accurate and reproducible gap measurements across multiple surgeons [11].

Ma et al. [12] report better short-term results and better planning with robot TKA than with conventional surgery. Moreover, the effect of PCL resection was investigated in the same patients, throughout flexion including midflexion, and with the patella reduced. Patellar eversion during gap measurements has been shown to significantly alter gap and ligament tension values, changing gaps by up to 3 mm and 5° [5, 13, 14].

There are several limitations to our study. First, our sample size was relatively small at 55 knees. This number is comparable to other clinical studies however which include sample sizes on the order of 17 [6], 30 [3, 15] and 50 [16] patients, and represents an improvement over cadaver studies which used only about ten to 12 specimens [2, 5]. While we analyzed differences between neutral/varus and valgus HKA angles, we did not have sufficient numbers to analyze for additional phenotype subgroups such as those classified by Hirschmann et al. [17]. Additional data would be required for this and future research on this may be warranted. We also used data from two surgeons and while the technique was standardized and both surgeons used the same robotic system to make the measurements before and after PCL resection, some slight variations in technique between surgeons may have been unavoidable. Peng and al [18] shown differences in the sagittal alignment of TKAs, between the axes referenced by robot-assisted TKA and those referenced by conventional TKA.

Additionally, while the tensioner used in this study allowed for the patella to be reduced during the acquisitions, the device did not allow for complete closure of the capsule as the arms of the distractor system were extending out from the wound. Patellar eversion vs complete closure of the joint capsule with sutures may result in some differences in the gap readings, albeit likely small. Finally, although we standardized the load applied to 80N per side throughout flexion, the ideal load to apply during ligament balancing in TKA is still debated and applying loads of different magnitude could change the gap openings and the difference in opening between the medial and lateral sides. One study reported the difference in flexion gaps when increasing load from 70 to 90N as 1 and 2 mm on the medial and lateral sides, respectively [19]. However, the 80N force applied in our study is commonly used and recommended for gap balancing in TKA in other studies [20–22].

## Conclusions

Resecting the PCL results in unequal and unsymmetric increases in tibiofemoral gaps, where the gap increase is negligible in extension and largest in flexion. Gap changes are dependent on pre-operative deformity, where valgus knees demonstrate larger increases in flexion laxity with PCL resection than neutral/varus knees, and have greater mediolateral differences in opening, with the lateral side opening more than the medial side. These results have direct implications on gap balancing in TKA. Surgeons should expect PCL resection to cause greater increases in laxity and in mediolateral imbalance in valgus vs neutral/varus knees, where valgus knees open up more laterally than medially in midflexion and flexion after PCL resection. If the PCL is intended to be resected, this should be performed prior to performing gap measurements and making bone resections to avoid having an overly large flexion gap after TKA.

## Data Availability

No datasets were generated or analysed during the current study.
